# The MAPP research network: design, patient characterization and operations

**DOI:** 10.1186/1471-2490-14-58

**Published:** 2014-08-01

**Authors:** J Richard Landis, David A Williams, M Scott Lucia, Daniel J Clauw, Bruce D Naliboff, Nancy A Robinson, Adrie van Bokhoven, Siobhan Sutcliffe, Anthony J Schaeffer, Larissa V Rodriguez, Emeran A Mayer, H Henry Lai, John N Krieger, Karl J Kreder, Niloofar Afari, Gerald L Andriole, Catherine S Bradley, James W Griffith, David J Klumpp, Barry A Hong, Susan K Lutgendorf, Dedra Buchwald, Claire C Yang, Sean Mackey, Michel A Pontari, Philip Hanno, John W Kusek, Chris Mullins, J Quentin Clemens

**Affiliations:** 1 Department of Biostatistics and Epidemiology, Perelman School of Medicine at the University of Pennsylvania, Philadelphia, PA, USA; 2 Departments of Anesthesiology, Medicine and Psychiatry, University of Michigan, Ann Arbor, MI, USA; 3 Department of Pathology, University of Colorado Anschutz Medical Campus, Aurora, CO, USA; 4 Departments of Medicine, Psychiatry, and Gastroenterology, University of California, Los Angeles, CA, USA; 5 Division of Public Health Sciences, Department of Surgery, Washington University, St Louis, MO, USA; 6 Department of Urology, Northwestern University, Chicago, IL, USA; 7 Department of Urology, University of Southern California, Beverly Hills, CA, USA; 8 Division of Digestive Diseases, University of California, Los Angeles, CA, USA; 9 Division of Urologic Surgery, Department of Surgery, Washington University School of Medicine, St. Louis, MO, USA; 10 Department of Urology, University of Washington, Seattle, WA, USA; 11 Department of Urology, University of Iowa, Iowa City, IA, USA; 12 VA Center of Excellence for Stress and Mental Health, University of California San Diego, San Diego, CA, USA; 13 Departments of Obstetrics and Gynecology, Urology and Epidemiology, University of Iowa, Iowa City, IA, USA; 14 Department of Medical Social Sciences, Northwestern University, Chicago, IL, USA; 15 Departments of Psychiatry and Medicine, Washington University School of Medicine, St. Louis, MO, USA; 16 Departments of Epidemiology and Medicine, University of Washington, Seattle, WA, USA; 17 Department of Anesthesiology, Division of Pain Medicine, Stanford University School of Medicine, Palo Alto, CA, USA; 18 Department of Urology, Temple University School of Medicine, Philadelphia, PA, USA; 19 Department of Urology, Perelman School of Medicine at the University of Pennsylvania, Philadelphia, PA, USA; 20 National Institute of Diabetes and Digestive and Kidney Diseases, National Institutes of Health, Bethesda, MD, USA; 21 Department of Urology, Division of Neurourology and Pelvic Reconstructive Surgery, University of Michigan, Ann Arbor, MI, USA

**Keywords:** Urologic chronic pelvic pain syndromes, Interstitial cystitis, Chronic prostatitis, Urine biomarkers, Plasma biomarkers, Non-urologic associated syndromes, Quantitative sensory testing (QST), Neuroimaging

## Abstract

**Background:**

The “Multidisciplinary Approach to the Study of Chronic Pelvic Pain” (MAPP) Research Network was established by the NIDDK to better understand the pathophysiology of urologic chronic pelvic pain syndromes (UCPPS), to inform future clinical trials and improve clinical care. The evolution, organization, and scientific scope of the MAPP Research Network, and the unique approach of the network’s central study and common data elements are described.

**Methods:**

The primary scientific protocol for the Trans-MAPP Epidemiology/Phenotyping (EP) Study comprises a multi-site, longitudinal observational study, including bi-weekly internet-based symptom assessments, following a comprehensive in-clinic deep-phenotyping array of urological symptoms, non-urological symptoms and psychosocial factors to evaluate men and women with UCPPS. Healthy controls, matched on sex and age, as well as “positive” controls meeting the non-urologic associated syndromes (NUAS) criteria for one or more of the target conditions of Fibromyalgia (FM), Chronic Fatigue Syndrome (CFS) or Irritable Bowel Syndrome (IBS), were also evaluated. Additional, complementary studies addressing diverse hypotheses are integrated into the Trans-MAPP EP Study to provide a systemic characterization of study participants, including biomarker discovery studies of infectious agents, quantitative sensory testing, and structural and resting state neuroimaging and functional neurobiology studies. A highly novel effort to develop and assess clinically relevant animal models of UCPPS was also undertaken to allow improved translation between clinical and mechanistic studies. Recruitment into the central study occurred at six Discovery Sites in the United States, resulting in a total of 1,039 enrolled participants, exceeding the original targets. The biospecimen collection rate at baseline visits reached nearly 100%, and 279 participants underwent common neuroimaging through a standardized protocol. An extended follow-up study for 161 of the UCPPS participants is ongoing.

**Discussion:**

The MAPP Research Network represents a novel, comprehensive approach to the study of UCPPS, as well as other concomitant NUAS. Findings are expected to provide significant advances in understanding UCPPS pathophysiology that will ultimately inform future clinical trials and lead to improvements in patient care. Furthermore, the structure and methodologies developed by the MAPP Network provide the foundation upon which future studies of other urologic or non-urologic disorders can be based.

**Trial registration:**

ClinicalTrials.gov identifier: NCT01098279 “Chronic Pelvic Pain Study of Individuals with Diagnoses or Symptoms of Interstitial Cystitis and/or Chronic Prostatitis (MAPP-EP)”. http://clinicaltrials.gov/show/NCT01098279

## Background

Interstitial cystitis/bladder pain syndrome (IC/BPS) and chronic prostatitis/chronic pelvic pain syndrome (CP/CPPS) are defined by the hallmark symptom of chronic pain in the region of the pelvis, urogenital floor, or external genitalia, often accompanied by urinary symptoms, such as urinary urgency or frequency [1,2]. The bladder has historically been thought to be the origin of IC/BPS symptoms; whereas the prostate gland has traditionally been believed to be the source of CP/CPPS symptoms. However, this viewpoint has come under recent challenge, in large part from the observation that many IC/BPS and CP/CPPS patients exhibiting symptoms do not have identifiable pathology in these organs [3-5].

The impact and burden of IC/BPS and CP/CPPS are substantial. Patients suffer considerable morbidity resulting in a significant decrease in quality of life for both the patient and his/her partner due to the physical and psychological impact of the condition. In fact, the quality of life of IC patients has been characterized as being worse than that of patients undergoing dialysis [6]. In the U.S., the prevalence of IC/BPS symptoms has been estimated to be 2.7% in women [7]; whereas the prevalence for analogous symptoms is estimated to be 1.3% in men [8]. Prevalence estimates for CP/CPPS in men vary between 1.8 - 6.4%, depending upon case definitions and screening methods [9,10].

Traditionally, the diagnosis of IC required cystoscopy and pathological findings, although more typically IC/BPS is defined from patient reported symptoms, due to the lack of consistent pathological findings, defined disease phenotypes or biological markers. Similarly, patient symptoms are used to define CP/CPPS. Each of these separate syndromes, however, may in fact represent a group of related conditions that manifest in a similar manner, but have differing etiologies. Based primarily on their somewhat similar symptom profiles [2], IC/BPS and CP/CPPS are here collectively termed urologic chronic pelvic pain syndromes (UCPPS) (see Table 1 for research definitions used in the MAPP Research Network). As noted in the only published phenotyping system, referred to as UPOINT [11,12], UCPPS patients have significant symptoms across the urinary, psychosocial, organ specific, infection, neurological/systemic and tenderness domains, confirming the heterogenous nature of these syndromes.

**Table 1:** Terminology used in the MAPP Research Network to described disorders under study

**Term**	**MAPP Network Research Definition**
Urologic chronic pelvic pain syndrome (UCPPS)	General term to describe idiopathic chronic pelvic pain of urologic origin in men or women. In MAPP Network studies, this includes men and women with IC/BPS, or men with CP/CPPS (see below).
Interstitial cystitis/bladder pain syndrome (IC/BPS)	Chronic unpleasant sensation (pain, pressure, discomfort) perceived to be related to the urinary bladder, associated with lower urinary tract symptoms, in the absence of infection or other identifiable causes.
Chronic prostatitis/chronic pelvic pain syndrome (CP/CPPS)	Chronic idiopathic pelvic pain or discomfort in males, commonly in the perineum, suprapubic region, penis, or testicles, which is often exacerbated by ejaculation or urination.
Non-Urologic Associated Syndromes (NUAS)	General term used to describe symptom-based non-urologic syndromes which co-occur with UCPPS at a rate greater than observed in the general population. Within the MAPP Network, initial efforts have focused on studying specific NUAS (fibromyalgia, irritable bowel syndrome, chronic fatigue syndrome), though other examples exist (e.g. vulvodynia, temperomandibular joint disorder, and migraine headaches, among others).

Despite intensive study over the past decade, clinical trials have failed to identify effective therapies, and basic science studies have failed to identify specific pathophysiology for these conditions (for a review of previous research efforts see companion Commentary by Clemens, et al) [13]. Intriguing new clues into the pathophysiology of UCPPS have come from several epidemiological studies revealing shared pathophysiology between UCPPS and other conditions having chronic pain as a cardinal or prominent symptom (e.g., fibromyalgia, irritable bowel syndrome, endometriosis, chronic fatigue syndrome, and vulvodynia) [14-20]. Some of these chronic pain conditions were previously thought to be due to “peripheral” etiologies (i.e., damage or inflammation in the region of the body where the individual was experiencing pain) but are now known to have prominent central nervous system (CNS) contributions (i.e. centralized pain). In light of this new understanding, even the names of some of these conditions have changed, reflecting the fact that peripheral inflammation is not the primary cause of symptoms (e.g., fibrositis became fibromyalgia, spastic colitis became irritable bowel syndrome). In a similar manner, it has been suggested that IC be renamed Bladder Pain Syndrome (BPS), based on the fact that the majority of patients do not have identifiable inflammation or even pathology in the bladder [21]. Collectively, these findings suggest the merits of exploring common underlying CNS pathophysiology (i.e., pain centralization or augmentation) [22-24] rather than continuing the search for a uniform malfunction of a single end organ [23].

In recognition of these emerging insights and the limitations of previous basic research and clinical studies, the NIDDK established the “Multidisciplinary Approach to the Study of Chronic Pelvic Pain” (MAPP) Research Network to better understand the etiology and treated natural history of UCPPS. This network holds the promise of advancing UCPPS treatment through the identification of clinically relevant patient subgroups potentially requiring distinct interventions [13]. By moving beyond traditional bladder- and prostate-focused investigations toward an innovative multidisciplinary research strategy, the MAPP Research Network is able to more fully investigate the relationship between UCPPS and non-urologic associated syndromes (NUAS), and better define urologic and more systemic contributions to the pathophysiology of these disabling syndromes.

This manuscript describes the approach to clinical phenotyping developed in the MAPP Research Network, with a focus on its central epidemiological study. This integrated research design is highly unique in its evaluation of visceral pain and lower urinary tract symptoms associated with UCPPS, and represents the largest and most detailed characterization of UCPPS to date.

## Methods/design

### Scientific focus

The MAPP Research Network conducts complementary basic, translational, and clinical science studies to investigate questions of clinical relevance, motivated by the view that UCPPS involves substantial central systemic mechanisms. Studies have been designed to advance our understanding of the underlying pathophysiology and etiology, treated natural history, “flare” etiology, risk factors associated with biologic, genetic, and behavioral factors, and the discovery of comprehensive characterizations of patient phenotypes. Another key objective has been to address the relationships between UCPPS and commonly associated non-urologic syndromes (Table 1). The MAPP Network also supports translational studies using UCPPS animal models founded on key clinical criteria and leading hypotheses of UCPPS etiology.

### MAPP network organization

**Figure 1:**
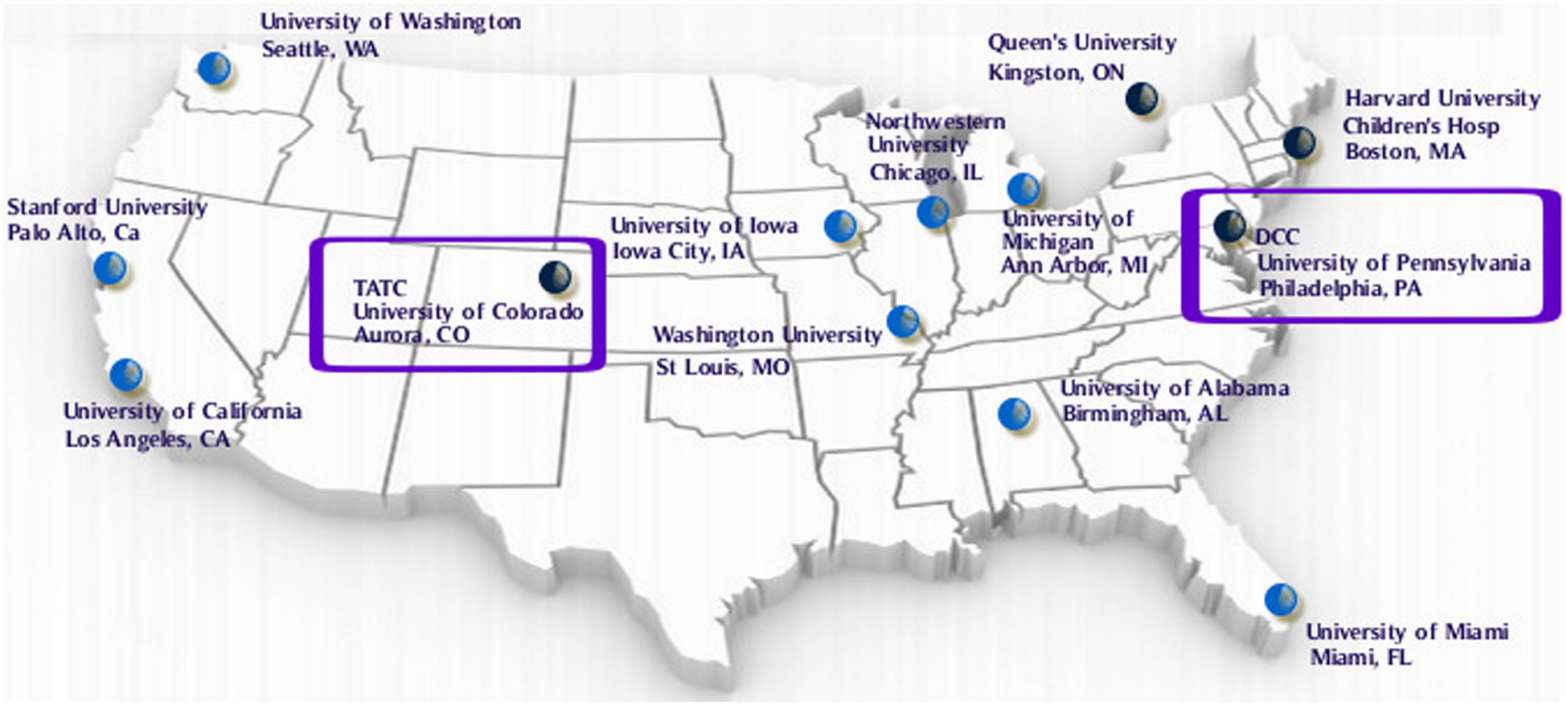
MAPP Research Network Discovery and Collaborating Sites, Data Coordinating Core (DCC) and the Tissue Analysis and Technology Core (TATC).

The MAPP Research Network consists of six discovery sites (Los Angeles, CA; Chicago, IL; St. Louis, MO; Iowa City, IA, Seattle, WA, and Ann Arbor, MI); several satellite sites (Miami, FL; Birmingham, AL; Palo Alto, CA; Boston, MA; Kingston, Ontario, Canada (CA); a data coordinating core (DCC) in Philadelphia, PA; a tissue analysis and technology core (TATC) in Aurora, CO; a neuro-imaging scan repository and reading center (Los Angeles, CA); an external experts panel (EEP); and NIDDK project scientists (Figure 1).

### MAPP network core functions

**Figure 2:**
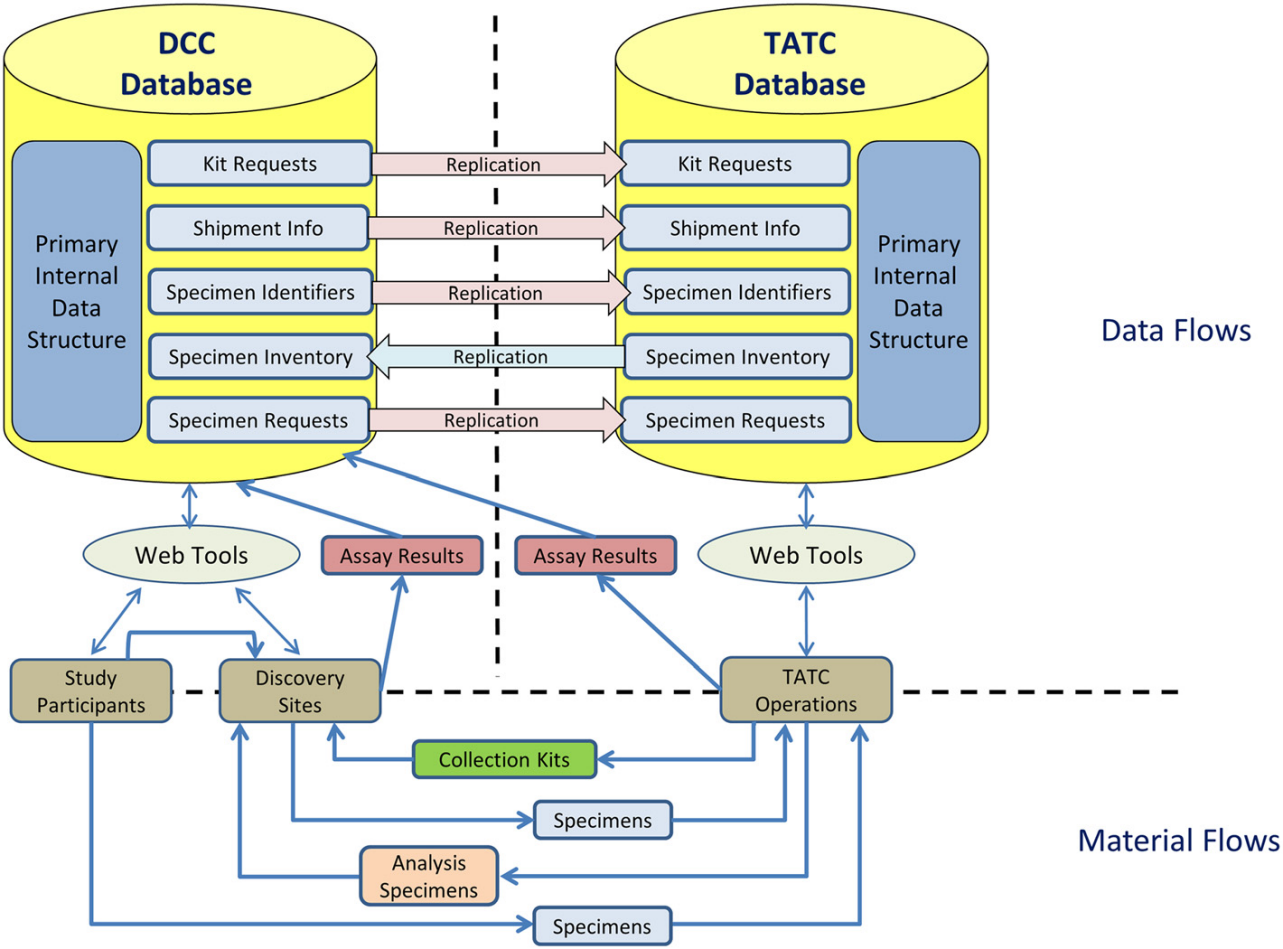
Data and Materials Flow Schematic: Left Panel (Data Coordinating Core (DCC) Database) and Right Panel (Tissue Analysis and Technology Core (TATC) Database).

The DCC provides biostatistical design and analysis leadership for all studies, serves as the central site for electronic protocol and data management system development, web-based deployment of data capture tools, data acquisition and storage, and promotes network-wide quality assurance across all protocol-specific domains of data. The DCC also provides administrative and project coordination support, including development and maintenance of a public website (http://www.mappnetwork.org/). The TATC provides a central location for bio-specimen processing, storage, and analysis of blood, urine, and DNA samples. The TATC established and implemented standards for specimen collection, identification, and handling to promote consistent specimen collection procedures. DCC and TATC personnel conducted centralized coordinator training before initiating patient enrollment, with follow-up re-fresher training at periodic Steering Committee meetings. Standardized modular barcoded collection kits with specimen annotation forms for blood, urine, and cheek swab DNA were designed for use at all recruitment sites. The data-sharing model allows sites to enter material requests, specimen collection and shipment information through the DCC portal, with replication in real-time between both DCC and TATC databases (Figure 2). Sites request kits via the DCC portal, following which the TATC sends a shipment of unlinked specimen kits, uniquely identified with a barcode system, to the site, which are individually linked through the DCC portal to a MAPP participant at the time of biospecimen collection. Blood specimens are shipped on the day of collection to the TATC for next day delivery; whereas cheek swab and urine specimens are temporary stored at the collection sites and batch-shipped to the TATC. At-home specimen collection is facilitated by participants, using barcoded collection materials and pre-labeled shipping containers for direct shipping to the TATC. Centralized processing at the TATC ensures standardized processing using best practice standards [25]. Derivative specimen aliquots are barcoded without participant identifying information, allowing for blinded discovery and validation projects. A rigorous series of identity management procedures were implemented between the DCC and TATC to ensure unambiguous links between specimen and participant data. Discovery sites submit specimen requests for research projects to the DCC. Sharing of real-time specimen inventory and annotation data between the TATC and DCC database allows full control by the DCC to match specimens with the required clinical data, and select specimens at the aliquot tube level. After selection by the DCC, a specimen distribution request is transferred to the TATC, triggering shipment to the discovery site. Real-time updates allow the DCC to monitor progress of distribution projects.

### Quantitative Sensory Testing (QST)

QST is a specialized form of assessment that utilizes Pressure Pain Threshold (PPT) to assess pain sensitivity to standardized evoked stimuli. The PPT was determined by using blunt pressure delivered by a 1-cm^2^ hard-rubber probe to the thumbnail bed of the participant’s non-dominant hand [26-28]. The thumbnail is considered a “neutral site”, both because it is distant from the pain that these individuals are experiencing, and this site has been repeatedly shown to be a good measure of overall “central” pain threshold [29]. Evoked pressure pain, rather than heat pain threshold testing was chosen for MAPP studies for several reasons; first, QST studies have found that pressure pain testing differentiates IC from healthy controls better than does heat stimulation [16,30,31], and secondly, pressure testing was more easily deployed in a standardized manner across multiple sites.

### Neuroimaging and functional neurobiology

Neuroimaging and functional neurobiology studies support overall participant characterization, as well as specific trans-MAPP brain imaging studies. These studies were designed to identify differences in brain structure and connectivity [32,33] as well as differences in regional intrinsic oscillation frequencies and resting state connectivity of the brain [34] between UCPPS patients and control groups. A standardized protocol was developed to acquire structural (grey matter), diffusion tensor imaging; white matter (integrity and connectivity) and resting state images of the brain, as well as functional neurobiology studies across study sites. The magnetic resonance imaging/functional magnetic resonance imaging (MRI/fMRI) data repository for this study was established at the UCLA Center for Neurobiology of Stress (http://pain.med.ucla.edu/), in close collaboration with UCLA-Laboratory of Neuroimaging (LONI), which has extensive experience in the collection, storage and analysis of large multi-site MRI data sets (loni.usc.edu) [35].

### Biomarker and infectious etiology studies

As a component of the Trans-MAPP EP Study protocol, biological samples (urine, plasma, and cheek swab DNA) were collected at Discovery Sites at baseline (UCPPS patients and control cohorts) and the 6- and 12-month in-clinic office visits (UCPPS patients) (see Table 2 for details on samples collected). In addition, a specialized single-use at-home collection kit was developed for use by UCPPS patients at the time of self-reported flare. These samples were archived at the TATC and distributed to MAPP Network investigators, in conjunction with associated clinical data managed at the DCC, to support integrated studies to identify and characterize UCPPS biological markers (biomarkers) and to examine the potential contributions of infectious agents to UCPPS. The biomarker study uses UCPPS and control plasma and spot-urine collections (Table 2) to assess the utility of previously identified, candidate markers and to identify new markers in a discovery-based approach using proteomics platforms. The infectious etiology study examines VB1 and VB2 (males and females) and VB3 (males) urine samples (Table 2) through advanced 16S deep sequencing methods to assess UCPPS and control microbial profiles and to address hypotheses regarding an infectious basis for symptom development and fluctuations, including symptom flare. Archived DNA samples will be used for targeted epigenetics and genetic investigations.

**Table 2:** Number of participants (target, enrolled) by cohort, sex (UCPPS: duration of symptoms), and number of participants with biospecimens by type, MRI scans completed and PPT data collected at baseline visit

**Cohorts**	**No. of participants by cohort**		**No. of participants with biospecimens**						**No. of participants with MRI scans**	**No. of Participants with Pain Pressure Threshold (PPT) measures**	
	**Target size**	**Actual enrolled**	**Cheek swab**	**Biomarker samples**		**Infectious etiology urine samples**				**At baseline**	**During follow-up**
				**Plasma**	**Urine**	**VB1**	**VB2**	**VB3**			
**UCPPS (Duration of symptoms)**											
***Male < 2 Yrs***	95	90	90	90	90	90	90	39	13	19	20
***Male ≥ Yrs***	95	101	100	100	101	100	100	42	22	12	20
***Female < 2 Yrs***	95	89	89	86	89	89	88	0	22	20	19
***Female ≥2 Yrs***	95	144	144	142	144	142	139	0	41	11	23
**Total**	**380**	**424**	**423**	**418**	**424**	**421**	**417**	**81**	**98**	**62**	**82**
**Healthy controls**											
***Male***	190	182	182	177	181	176	174	64	38	40	0
***Female***	190	233	233	228	232	232	227	0	79	60	0
**Total**	**380**	**415**	**415**	**405**	**413**	**408**	**401**	**64**	**117**	**100**	**0**
**Positive controls**											
***Male***	95	44	44	43	43	43	43	10	17	8	0
***Female***	95	156	156	154	154	154	152	0	47	27	0
**Total**	**190**	**200**	**200**	**197**	**197**	**197**	**195**	**10**	**64**	**35**	**0**
**Overall total**	**950**	**1,039**	**1,038**	**1,020**	**1,034**	**1,026**	**1,013**	**155**	**279**	**197**	**82**

### Flare assessment in UCPPS

Currently it is unknown what triggers a flare of UCPPS symptomatology. Putative risk factors for flares (e.g., dietary factors, physical activity, stress, sexual activities, recent infections) were assessed for participants reporting a flare (limited to three assessments for subjects reporting >3 flares). The same potential flare risk factors were also collected during randomly selected follow-up contacts for participants when not reporting flares, serving as within-person control data. A home collection kit was designed to allow participants to collect urine samples for biomarker and infectious etiology studies at their first reported “flare”. In addition, each participant collected a reference urine sample using the home collection kit at one of the three randomly selected non-flare time points during the first four months. A full set of flare and non-flare biomarker and infectious etiology urine specimens was successfully collected from 188 (44%) of the participants. Another 44 (10%) participants provided only a flare specimen set; whereas 123 (29%) of participants provided only a non-flare specimen set. This reference specimen, together with the urine specimens collected during the in-clinic visits will be analyzed, and compared with the flare specimen, to investigate potential biomarkers and infectious agents that are uniquely present in urine during symptom flares.

### Baseline self-report battery characterizing UCPPS and controls

Extensive clinical phenotyping was conducted to characterize UCPPS patients, positive controls and healthy controls at baseline. A description of the self-report battery follows categorized by: General Socio-Demographic and Medical History; Urological Specific Measures; and Non-Urological co-morbidities and diagnostics.

#### ***General socio-demographic and medical history***

Data on age, sex, race/ethnicity, education, marital status, and income were collected from participant self-report. A directed medical history was gathered on each participant that included co-morbid conditions, early life infection history, concomitant medication use (i.e., name, dose, frequency and route of administration), previous treatments for UCPPS, and family medical history (i.e., history of chronic pain and psychiatric disorders in parents, grandparents, aunts, uncles, siblings, and children). A physical examination included measurement of height, weight and blood pressure as well as abdominal, pelvic, and rectal examinations. Pelvic floor muscle tenderness (yes/no) and suprapubic tenderness (yes/no) were evaluated in each participant. In women, the presence and degree of pelvic organ prolapse was recorded (above or below the hymenal ring). In men, penile exam (circumcised or not), prostate examination (enlarged, irregular, tender) and scrotal examination (varicocele, hydrocele, mass, hernia) findings were recorded.

#### ***Urological specific measures***

Urological measures were selected purposefully to provide continuity between MAPP and pre-existing literatures on IC/BPS and IC/CPPS. The urological measures thus sought to assess symptoms that have been historically considered relevant to UCPPS with instruments designed specifically for this population (Table 3).

##### 

**Symptom and health care utilization Questionnaire (SYM-Q)** A 12-item questionnaire was developed specifically for this study. All participants completed this questionnaire at baseline and at bi-weekly intervals throughout the 48-week study period. The SYM-Q inquires about (1) pain, urgency, frequency, (2) the presence of non-urological pain symptoms, (3) mood, (4) health care seeking, (5) last menstrual period, and (6) the occurrence of symptom worsening (flares).

##### 

**Interstitial Cystitis Symptom Index (ICSI) and problem index (ICPI) (ICINDEX)** This instrument consists of two 4-item questionnaires. The ICSI quantifies urinary and pain symptoms in patients with IC/BPS, and the ICPI assesses the degree of bother associated with these symptoms [36].

##### 

**American Urological Association Symptom Index (AUASI)** This validated 8-item questionnaire assesses the severity of voiding symptoms (e.g., frequency, urgency, nocturia, sense of incomplete emptying, intermittency, slow stream, straining to void) and associated bother in either sex [37].

##### 

**Brief Flare Risk Factor Questionnaire (BFRFQ)** Developed specifically for use in the MAPP Research Network as a means of better understanding flares in UCPPS, the BFRFQ contains 33 items documenting potential causes of flares. It includes questions about diet, physical activity, stress, sexual activity, and infection.

##### 

**Rand Interstitial Cystitis Epidemiology (RICE) case definition** The 5-item RICE case definition questionnaire was designed for epidemiological studies to identify the presence of IC/BPS symptoms in men and women [9]. We used it to identify sub-groups with or without bladder pain and urgency due to pain, pressure, or discomfort.

##### 

**Genitourinary Pain Index (GUPI)** This 9-item instrument was developed by modifying the original NIH-Chronic Prostatitis Symptom Index (CPSI) [38]. Several new items about bladder-specific pain were added, and male gender-specific items were replaced with female-gender specific items for women. The GUPI is applicable to men and women to assess pain symptoms, urinary symptoms, and quality of life as separate sub-scales, and overall as a total score [39].

##### 

**Female Sexual Function Index (FSFI)** This 19-item scale provides an overall assessment of female sexual functioning over the past 4 weeks, and includes domain scores in six areas: sexual desire, arousal, lubrication, orgasm, satisfaction, and pain, as well as a total score for sexual dysfunction [40].

##### 

**International Index of Erectile Function (IIEF)** The IIEF is a multidimensional instrument consisting of 15 items and 5 domains of male sexual function: erectile function, orgasmic function, sexual desire, intercourse satisfaction, and overall satisfaction. Total scores indicate greater levels of sexual dysfunction [41].

##### 

**Washington Male sexual function scale (MSFS)** This instrument was used in males to rate the severity of the following symptoms: pain with ejaculation, lack of interest in sexual activity, premature ejaculation, and difficulty reaching ejaculation [42].

##### 

**Self-Esteem And Relationship questionnaire (SEAR: M & F)** The SEAR is a 14-item measure validated to assess psychosocial impact of erectile dysfunction. The measure assesses an overall score, sexual relationship satisfaction, confidence, and self-esteem. The measure was adapted for use in women by omitting the two male-specific items [43].

**Table 3:** Baseline phenotyping battery for MAPP: urological self-report questionnaires

**Instrument**	**Subscales**
Symptom and Health Care Utilization Questionnaire (SYM-Q)	1. Pain, urgency, frequency
	2. Urologic/Pelvic Pain severity
	3. Non-urologic/Pelvic Pain severity
	4. Mood
	5. Most bothersome symptom
	6. Medical care seeking
	7. Menstrual information
	8. Flare status
Interstitial cystitis symptom and problem index	1. IC Symptom Index (ICSI)
	2. IC Problem Index (ICPI)
American Urological Association Symptom Index Score	1. AUASI total score
Rice case definition questionnaire	1. RICE total score
Brief flare risk factor questionnaire	1. Flare timing, symptoms, and symptom severity
	2. Cause attribution
	3. Foods
	4. Drinks
	5. Physical and sedentary activities
	6. Stress
	7. Sexual activity
	8. Infections
Genitourinary Pain Scale (GUPI)	1. Pain
Male version	2. Urinary symptoms
Female version	3. Quality of life
	4. Total
Female sexual function index	1. FSFI total score
International index of erectile function	1. IIEF total score
University of Washington male sexual function scale	1. Pain with ejaculation
	2. Premature ejaculation
	3. Difficulty reaching ejaculation
Self-esteem and relationship questionnaire	1. SEAR total score
Males	
Females	

### Non-urologic co-occurring symptoms and diagnostics

Although pain is experienced locally at a site of discomfort, it is often accompanied by other symptoms that influence pain processing, modulation, and ultimately how it is experienced. Understanding these other influential factors can provide insight into the mechanisms associated with the etiology and maintenance of chronic pain. Thus, the following symptoms and traits were assessed in each participant (Table 4).

#### 

**History of co-occurring somatic symptoms** The Complex Multi-Symptom Inventory (CMSI) [44] is a 41-item symptom checklist of past year illnesses specific to functional syndromes (e.g., fibromyalgia (FM), chronic fatigue syndrome (CFS), irritable bowel syndrome (IBS), vulvodynia (VVD), migraine, and temporomandibular disorders (TMD). The checklist provides a proxy for the presence of these comorbid conditions; whereas the follow–up assessment module uses the standardized diagnostic criteria for each condition [44]. The summed checklist has been interpreted as representing the overall symptom burden from somatic or functional conditions.

#### 

**Clinical pain** The Brief Pain Inventory (BPI) is a 15-item self-report measure that has been validated for use in a wide variety of pain states [45]. The BPI assesses for the presence of pain, pain intensity (i.e., worse, least, average, current), and functional interference from pain. It also catalogues the types of medications being used; the percentage of pain relief obtained from medications, and assesses pain distribution (via a body map). For purposes of the MAPP Research Network, the body map of the BPI was replaced with a more detailed body map used in epidemiological studies to better identify widespread pain by body regions [46]. This more detailed body map was further modified to include larger scale depictions of the pelvic and genital regions. This body map was scored to identify those where pelvic pain was confined to the pelvic region (i.e., pelvic pain only) or was more widespread extending beyond the pelvic region (i.e., pelvic pain and beyond).

#### 

**Functional status** The SF-12 [47], is a 12-item measure of functional status and generic quality of life. The instrument assesses eight domains of health status: physical functioning, role limitations because of physical problems, bodily pain, general health perceptions, energy/vitality, social functioning, role limitations due to emotional problems, and mental health. It also provides both a composite physical functioning score (PCS) and a mental health composite score (MCS). Higher scores on these measures indicate better functioning within the domain.

#### 

**Fatigue and sleep disturbance** The NIH Patient Reported Outcomes Measurement Information System (PROMIS) developed questionnaires for fatigue and sleep disturbance that can be used across disease conditions. Participants completed the following PROMIS short-forms: Fatigue (7-items) and Sleep Disturbance (8-items) [48]. Higher scores on these measures indicate worse symptomatology.

#### 

**Cognitive difficulties** The Multiple Ability Self-Report Questionnaire (MASQ) assessed the self-perception of having cognitive difficulties [49]. This is a 38-item questionnaire comprised of 5 domains of cognitive concerns: language ability, visio-spatial ability, verbal memory, visual memory, and attention/concentration. Validation studies have found the self-reported cognitive difficulties to correspond to performance-based indices of the same constructs [49]. Higher scores on each scale indicate more problematic perceptions.

#### 

**Stress** Perceived stress was measured using the 10-item Perceived Stress Scale (PSS) [50]. The PSS measures the degree to which situations are perceived as being unpredictable, uncontrollable and overwhelming. Higher scores indicate more stress.

#### 

**Emotional distress** Emotional distress was assessed across several affective domains. Depressive and anxiety-related symptoms were assessed using the Hospital Anxiety and Depression Scale (HADS) [51]. The HADS is a 14-item self-report questionnaire developed for use in non-psychiatric settings. This instrument provided both a depressive symptom and anxiety symptom score with validated cut-off scores associated with clinically relevant levels of each affective domain. Anger was assessed with the 8-item anger short form from PROMIS [48]. A potential source of resilience, (i.e., positive affect), was assessed using the Positive and Negative Affect Schedule (PANAS) [52]. This 20-item questionnaire provides both a positive and negative affect score. On each mood measure, higher scores indicate greater problems with mood.

#### 

**Personality traits** Personality was measured using the International Personality Item Pool (IPIP) short form [53]. The IPIP is a public-domain, 120-item instrument that was developed to reflect assessment of five personality domains: extraversion, neuroticism, agreeableness, conscientiousness, and openness to experience. Higher scores indicate greater strength of each personality trait.

#### 

**Catastrophizing** Catastrophizing refers to the perception that pain is overwhelmingly awful and the worst imaginable burden that one can endure. This cognitive perception was assessed using the 6-item Catastrophizing sub-scale from the Coping Strategies Questionnaire (CSQ) [54] a metric for measuring this construct. Higher scores indicate greater catastrophizing.

#### 

**Locus of control** The Beliefs in Pain Control Questionnaire (BPCQ) [55] is a 13-item questionnaire designed to evaluate beliefs regarding whether pain is under personal control or under the control of forces external to the patient. Three scales can be derived: (1) Internal scale – measuring beliefs that pain can be personally controlled (2) Powerful Doctors – an external locus of control scale measuring beliefs that pain control is in the hands of powerful others, and (3) Chance Happenings – a second external locus of control scale measuring beliefs that pain is controlled by chance or misfortune. Higher scores on each scale indicate greater strength of each belief.

#### 

**Early life trauma history** The Childhood Traumatic Events Scale (CTES) [56] is composed of two forms. The first assesses childhood traumatic events that occurred prior to the age of 17. Domains include death of a close family member or friend, parental separation, physical abuse including sexual assault, serious illness, and other. For each question, the age of trauma, perceived intensity of the trauma, and whether or not confiding in others occurred is assessed. The second form is labeled Recent Traumatic Events Scale (RTES). It assesses essentially the same traumatic domains with the exception that the timeframe is within the last 3 years, parental separation is replaced with spouse or significant other separation, and a new category of job change is added. This instrument can be scored to identify the intensity of each type of trauma or intensities can be summed across all traumas. This form is unique in that it also assesses whether the person confided in another individual about the trauma.

**Table 4:** Baseline phenotyping battery for MAPP: non-urological self-report questionnaires

**Instrument**	** Subscales**
Complex Multi-Symptom Inventory (CMSI) (Diagnostics of co-morbid functional disorders and overall symptom burden)	1. Fibromyalgia
	2. Chronic fatigue syndrome
	3. Irritable bowel syndrome
	4. Vulvadynia
	5. Migraine
	6. Temporomandibular disorders
	7. Past year total symptom burden
Brief Pain Inventory (BPI) (General clinical pain)	1. Severity
	2. Interference
	3. Medications
	4. Relief from medications
	5. Body map: overall
	6. Body map: male genital
	7. Body map: female genital
Short Form-12 (Functional status)	1. Physical status
	2. Physical role status
	3. Bodily pain
	4. General health
	5. Energy/vitality
	6. Social functioning
	7. Mental health
	8. Role limitations (emotional)
	9. Composite physical (PCS)
	10. Composite mental health (MCS)
PROMIS: Fatigue	1. Total score (t-score)
PROMIS: Sleep disturbance	1. Total score (t-score)
Multiple Abilities Self-Report Questionnaire (MASQ) (Perceived cognitive problems)	1. Language ability
	2. Visio-spatial ability
	3. Verbal memory
	4. Visual memory
	5. Attention/concentration
Perceived Stress Scale (PSS)	1. Total score
Hospital Anxiety and Depression Scale	1. Depressive symptoms (HADS:D)
	2. Anxiety Symptoms (HADS:A)
PROMIS: Anger	1. Total score (t-score)
Positive and Negative Affect Scale (PANAS)	1. Positive affect
	2. Negative affect
	3. Affect balance
International Personality Item Pool (IPIP)	1. Neuroticism
	2. Extroversion
	3. Agreeableness
	4. Conscientiousness
	5. Openness to experience
Coping Strategies Questionnaire (Catastrophizing scale)	1. Cat score
Beliefs in Pain Control Questionnaire (BPCQ)	1. Internal locus of control
	2. Powerful doctors (external locus)
	3. Chance (external locus)
Childhood Traumatic Events Scale (CTES) Recent Traumatic Events Scale (RTES)	1. Age of each trauma
	2. Intensity of each trauma
	3. Confiding in others for each trauma

### The trans-MAPP epidemiology/phenotyping study

**Figure 3:**
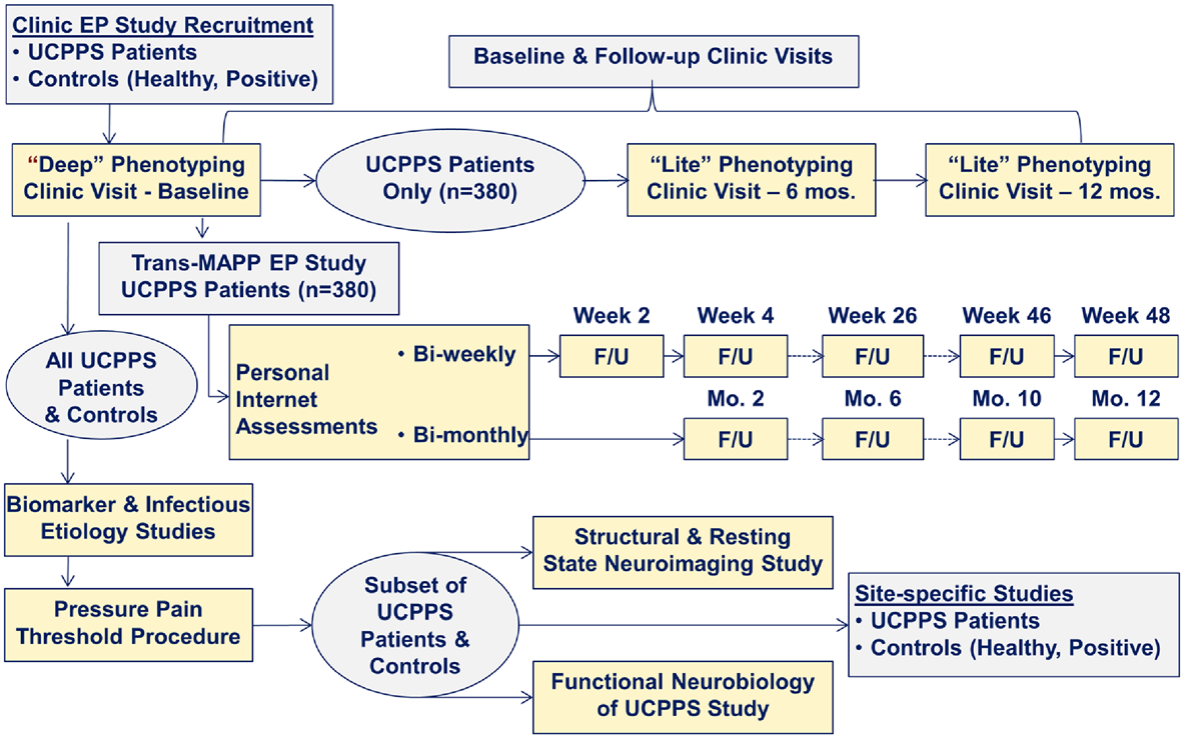
Trans-MAPP Epidemiology and Phenotyping (EP) Study of UCPPS Patients, Control (Healthy, Positive) Groups, Biomarker and Infectious Etiology Studies, Pressure Pain Threshold Study, Structural and Resting State Neuroimaging Study, and Functional Neurobiology of UCPPS Study.

The principal study conducted by the MAPP Research Network is a prospective observational study of the treated natural history of UCPPS - the Trans-MAPP Epidemiology/Phenotyping (EP) Study (Figure 3). This study serves as the central clinical phenotyping effort for all MAPP Network participants, and the platform for additional, integrated and complementary phenotyping efforts.

### Trans-MAPP EP study participants

As illustrated in Figure 3, the Trans-MAPP EP Study specifically targeted the recruitment of 380 UCPPS participants for “phenotyping” at baseline and for follow-up. An additional 380 healthy controls matched on sex and age, and 190 “positive” controls, meeting the criteria for one or more of the targeted NUAS were also recruited and phenotyped at baseline. In addition to the self-reported characterization of all study participants at baseline, a standardized protocol for the collection of biological samples (cheek swabs, plasma, urine) was implemented for additional multi-site biomarker and infectious etiology studies, quantitative sensory testing was used to measure pressure pain threshold (PPT), flares were assessed, and structural and resting state neuroimaging and functional neurobiology studies were also conducted to characterize UCPPS patients and controls.

The original recruitment targets were 50% males and 50% females, for both UCPPS and control cohorts. In addition, 50% of both male and female UCPPS participants were targeted to have recent onset of chronic pelvic pain symptoms (operationalized as < two years) and 50% longer symptom duration (operationalized as ≥ two years). Consequently, the recruitment target sample size was 95 for each of these four UCPPS participant subgroups (Table 2). The healthy control group targets were 190 males and 190 females, aiming to balance age and race/ethnicity distributions within the UCPPS subjects. The targets for positive controls were not specified, except for a total of 95 males and 95 females with one or more NUAS.

#### ***UCPPS Inclusion criteria***

Inclusion criteria for UCPPS participants were: 1) a diagnosis of IC/BPS or CP/CPPS, with urologic symptoms present a majority of the time during any 3 of the past 6 months (CP/CPPS) or the most recent 3 months (IC/BPS); 2) at least 18 years old; 3) reporting a non-zero score for bladder/prostate and/or pelvic region pain, pressure or discomfort during the past 2 weeks; and 4) consented to provide a blood or cheek swab sample to test DNA for genes related to the main study goals.

#### ***UCPPS exclusion criteria***

Exclusion criteria for UCPPS consisted of the following: symptomatic urethral stricture, on-going neurological conditions affecting the bladder or bowel, active autoimmune or infectious disorders, history of cystitis caused by tuberculosis or radiation or chemotherapies, history of non-dermatologic cancer, current major psychiatric disorders, or severe cardiac, pulmonary, renal, or hepatic disease. In addition, males diagnosed with unilateral orchalgia without pelvic symptoms, and males with a history of microwave thermotherapy, trans-urethral or needle ablation or other specified prostate procedures were excluded.

#### ***Eligibility criteria for controls***

To ensure a clearly-defined healthy control subgroup, potential control participants were excluded if they reported any pain in the pelvic or bladder region or chronic pain in more than one non-urologic body region. Like healthy controls, positive controls needed to be free of pain in the pelvic region, but also needed to qualify on the CMSI as having one of the targeted co-morbid conditions.

### Screening and baseline procedures

**Figure 4:**
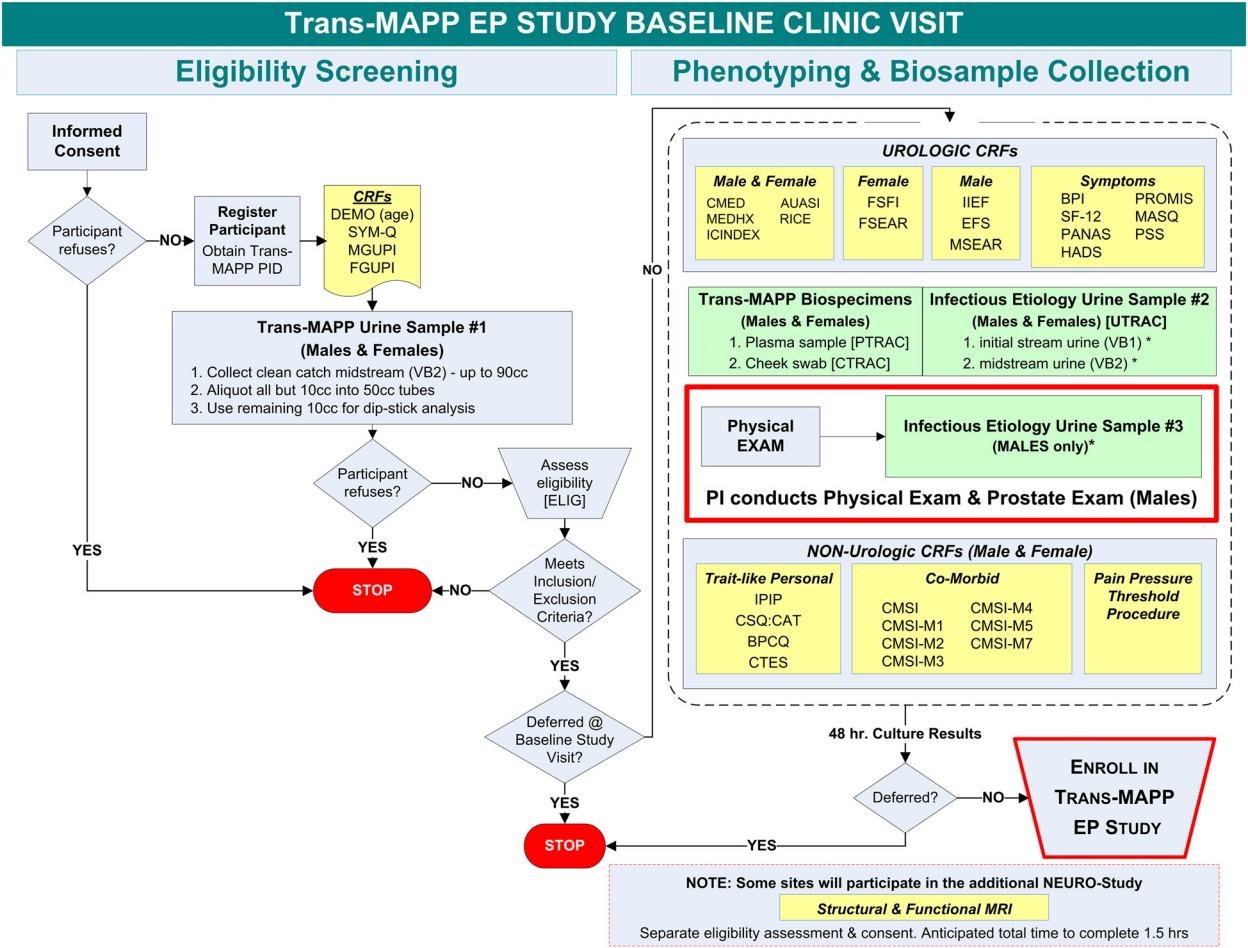
Sequence of Trans-MAPP Epidemiology and Phenotyping (EP) Study Screening, Phenotyping, Biospecimen Collection and Enrollment into the EP Study.

The screening and enrollment process utilized one in-clinic baseline study visit for informed consent and eligibility confirmation. This baseline visit was structured so that essential information, such as brief symptoms analogous to those used in previous UCPPS clinical studies (e.g., pain, pressure, discomfort and sex-specific symptom criteria) [57,58] and a urine sample dipstick could be acquired prior to the conduct of more intensive, invasive and time-consuming procedures (Figure 4). Persons meeting initial eligibility were then invited to complete the Trans-MAPP EP Study assessments, and were enrolled only after a negative 48-hour urine culture report. Eligible participants then underwent extensive baseline characterization using the standardized battery of urologic and non-urologic assessment instruments described previously. Biosamples, QST, and neuroimaging was also collected concurrently with the self-report information at baseline. Healthy controls, as well as “positive” controls (i.e., individuals with one or more non-urologic associated syndromes of primary interest; Table 2), were also enrolled but only underwent a single phenotyping assessment and biosample collection at baseline identical to that of participants with UCPPS.

### Longitudinal phenotyping schedule

After the extensive baseline assessment, participants were administered a small subset of the assessment battery for 48 weeks via internet-based bi-weekly / bi-monthly assessment questionnaires. More extensive assessment was possible at the in-clinic visit occurring at 24 and 48 weeks. Biological samples (e.g., DNA, serum, and urine) were collected in-clinic at baseline and at 24 and 48 weeks, as well as through home collection kits when patients reported symptom worsening (“flares”). The sequence of data and biospecimen collection is illustrated for the Trans-MAPP EP Study in Figure 4.

### Recruitment and retention of participants

Participant recruitment was conducted through the urology/urogynecology clinics at each of the clinical sites from 12/14/2009 through 12/14/2012. As summarized in Table 2, the MAPP Research Network enrolled 1,039 men and women, including persons with UCPPS (n = 424); “positive controls” with other NUAS (n = 200 for all conditions); and healthy controls (n = 415), exceeding specified target sizes. Given the success of recruitment and value of this phenotypic data, individuals with UCPPS will continue to be recruited and characterized in this manner in the subsequent phases of the network.

During the follow-up period, retention was promoted by the research coordinators by sending reminder messages to participants to provide their bi-weekly symptom assessments. Participants who missed these assessments were contacted by phone for further prompting to facilitate ongoing participation. The MAPP Research Network continued follow-up visits through 12/14/2013. The participant retention rate for the final clinic visit at 48 weeks was 83% (349/420), with remarkable adherence (83% missing no more than 3 monthly contacts). Among the 349 UCPPS participants completing the 48-week assessment, 161 (46%) to date have agreed (with re-consent) to participate in an ongoing extended follow-up study with internet-based symptom assessment every 4 months.

### Self-report and QST data

Currently, extensive baseline self-report phenotypic data is available on 424 individuals with UCPPS, 200 positive controls, and 415 healthy controls. Longitudinal assessment from all UCPPS participants was initiated with bi-weekly internet-based data capture, with 349 participants completing the 48 week final clinic visit. QST studies were performed on a subset of trans-MAPP EP participants from each site (n = 279) [59].

### Biological specimen collection

The biospecimen capture rate at the baseline visit was high, exceeding 98% for the cheek swabs, plasma and urine biomarker samples, and the first-void and infectious etiology mid-stream urine samples (VB1/VB2), as shown in Table 2. Success can be attributed to the tight integration of clinical procedures and biospecimen collection, the design of standardized collection kits, and the data sharing procedures (Figure 2) between the DCC and the TATC. In addition, consistent communication with research coordinators and monthly conference calls developed a strong collaborative environment, promoting timely response to data collection issues. The biosamples are examined in a set of integrated research protocols within the MAPP Network.

### Statistical considerations

All data are initially examined using exploratory descriptive methods, investigating potential differences between males and females, and between groups (i.e., UCPPS, healthy and positive controls). Categorical variables, including dichotomous factors, are summarized by proportions and compared among groups using standard chi-square tests of association and generalized Mantel-Haenszel methods, as described in Landis *et al. *[60].

Extensive baseline phenotype data are being investigated within a multi-stage cluster analysis [61] to construct clinically relevant subgroups, using a distance-dissimilarity matrix and clustering subjects using the average linkage method [62]. For each domain, the variables that are contributing most to the differences among the domain-specific clusters are identified.

Studies aimed at characterizing symptom pattern change over time utilize standard methods for longitudinal data analysis. For measured continuous outcomes, the primary models used are random coefficient growth curve models in which random effects due to subject and/or time are included to account for the correlation among repeated observations on each subject [63,64]. For binary or ordinal outcomes, the methodology of generalized estimating equations are being implemented to evaluate changes over time via logistic models [65,66].

To adjust for regression-to-the-mean effects, data from a pseudo-run-in period from baseline, 2 and 4 weeks of follow-up are being used to construct a baseline measure of variability, and a within-person slope is estimated beginning at 4 weeks through the final visit at 48 weeks. Studies aimed at evaluating factors related to the extent of symptom variability over time are being conducted utilizing subject-specific estimates of mean squared error about the estimated slope of longitudinal symptom outcomes.

## Discussion

The MAPP Research Network overall research strategy, and specifically its primary clinical protocol, the Trans-MAPP EP Study, represents a novel investigative direction for the field. Moreover, the integrated approach to systemic phenotyping allows a wealth of clinically important questions to be addressed. Through the use of state-of-the-art research methods developed by network investigators, and adopted from the broader pain field, the MAPP Network seeks to determine if UCPPS patients exhibit similar findings as have been observed for patients with non-urologic chronic pain. The recruitment of a healthy control group (individuals without urologic pain symptoms) and a “positive” control group (those with chronic non-urologic associated functional pain syndromes) allow MAPP Network investigators to search for novel clinical and biological measurements that may be unique to UCPPS, or that define clinically distinct UCPPS subgroups. The bi-weekly longitudinal assessments provide valuable information about UCPPS symptom variability and flares and, indeed, this effort has forged new ground in the highly effective acquisition of extensive, longitudinal clinical data through internet-based platforms developed specifically for this study. These extensive data collection efforts described here were developed to address a series of overarching study hypotheses prioritized by the MAPP Network for their clinical significance and relevance to advancing clinical care for UCPPS patients:

1. Individuals with UCPPS, measured at baseline and followed longitudinally for one year, as well as asymptomatic and disease comparator controls (e.g., CFS, FM, IBS) measured only at baseline, will make it possible to identify biologically-derived UCPPS subsets of individuals with UCPPS who: (a) have differing underlying pathogenesis resulting in their symptoms, and (b) would likely respond to different treatments.

2. There are two subsets of UCPPS patients: those with primarily pelvic symptoms, and those who also display many non-urological symptoms and syndromes. These latter individuals have a more systemic condition, characterized by a different natural history than those with isolated UCPPS symptoms, including a higher likelihood of: (a) symptom progression or continuation, (b) symptom variability, and (c) decreased quality of life and increased healthcare seeking behavior than those with primarily pelvic symptoms.

3. Individuals with UCPPS who have been symptomatic for longer periods of time (operationalized as 2+ years) will have more severe overall symptoms, decreased quality of life, and greater psychological co-morbidities than individuals with more recent (<2 years) onset of symptoms.

4. IC/BPS in females and CP/CPPS in males represent the same underlying condition. Using common phenotyping protocols in males and females, the high rate of non-urological symptoms and syndromes (NUAS) noted previously in women with IC/BPS will also be noted in men with CP/CPPS.

5. A variety of stressors (e.g, dietary, infectious, psychological) will be shown in case-crossover studies to predict worsening of symptoms (flares). Biomarker studies performed during these flares will identify factors in urine that increase with disease activity and decrease during quiescent periods.

6. Groups of individuals with UCPPS exhibit a lower overall pain threshold (i.e., hyperalgesia) compared to asymptomatic controls. This left-shift in stimulus-response function in the entire group of UCPPS patients will be noted, both on quantitative sensory testing, as well as within functional neuroimaging. This finding in the entire group of UCPPS patients will be shown to be driven by the subset of UCPPS patients with the more “systemic” form of the disease noted in Hypothesis 2 above.

7. Specific objective abnormalities (i.e., potential biomarkers, including central pain processing and modulation patterns (“brain signatures”) (Hypothesis 6) can be identified that are associated with specific risk factors (Hypothesis 5), reflecting specific pain processing and functional neuroimaging patterns (Hypothesis 6).

8. Disease development in subsets of UCPPS patients’ results from an underlying pathogenic process, and symptom exacerbations (flares) may be influenced by changes in pathogen type or quantity.

The phenotyping approach developed in the MAPP Network is the most comprehensive attempt to characterize individuals with UCPPS to date, and one of the most comprehensive phenotyping projects undertaken for any form of chronic pain. Examining not only urological factors, but also non-urological factors consistent with the biopsychosocial model of understanding chronic pain, broadens our ability to explore underlying pathological mechanisms that extend beyond the region of the pelvis. The observed variability across multiple domains within UCPPS suggests the possibility of clinically relevant subgroups that may be used to guide treatment in the future, or be used to understand different causal factors of pain and urinary symptoms. In addition, it is expected that future psychometric analyses will help to translate this extensive phenotyping battery into a set of assessment tools that can be applied in clinical practice so as to guide treatment decisions.

## Conclusions

The MAPP Research Network has successfully designed and implemented a 48-week longitudinal, observational study, focused on providing insights into underlying pathophysiology and identifying clinically relevant phenotypes of UCPPS. Healthy control subjects, and positive controls exhibiting NUAS, were also phenotyped to generate comparative data. Both baseline and longitudinal data collection is complete for the Trans-MAPP EP Study, which met or exceeded the original recruitment targets. Protocol adherence proved excellent, especially participant data collection directly through novel internet-based platforms. The biospecimen capture rate reached nearly 100%, and the participant retention rate for the final clinic visit at 48 weeks was 83% (349/420), with remarkable adherence (83% missing no more than 3 monthly contacts). These statistics reveal critical successes in the design and implementation of this complex, multi-site clinical study. A comprehensive, multidimensional battery of self-report measures is at the center of the clinical phenotyping. This was assembled by network investigators and incorporated into the Trans-MAPP Network EP Study to correlate an array of domains, including urologic symptoms, non-urologic pain, psychosocial factors, risk factors, quality of life, among others, in patients with UCPPS. Complementary data from integrated multi-site biomarker studies, neuroimaging, experimental pain testing, and a number of additional efforts were collected and are being used to further inform on pathophysiology and segregate clinically relevant subgroups of individuals with UCPPS, for whom diagnostics, treatments, and further studies of underlying pathological mechanisms can be targeted. These complex data are currently being analyzed to provide a systemic assessment of UCPPS patients and patient groups.

It is expected that information obtained from these studies will significantly aid in our understanding of the pathophysiology and clinical characteristics of UCPPS, inform future clinical efforts, and improve symptom management. Finally, the MAPP Research Network serves as a new model for how large multi-disciplinary efforts may be designed to investigate complex urologic, as well as non-urologic, conditions.

## Abbreviations

AUASI: American urological association symptom index; BACH: Boston Area Community Health Survey; BFRFQ: Brief flare risk factor questionnaire; BPCQ: Beliefs in Pain Control Questionnaire; BPI: Brief pain inventory; BPS: Bladder Pain Syndrome; CFS: chronic fatigue syndrome; CNS: Central nervous system; CPC: Chronic Prostatitis Cohort; CP/CPPS: Chronic prostatitis/chronic pelvic pain syndrome; CSQ: Coping Strategies Questionnaire; CTES: Childhood Traumatic Events Scale; DCC: Data coordinating core; DNA: Deoxyribonucleic acid; EEP: External experts panel; FM: Fibromyalgia; FSFI: Female sexual function index; GUPI: Genitourinary pain index; HADS: Hospital Anxiety and Depression Scale; IBS: Irritable bowel syndrome; IC/BPS: Interstitial cystitis/bladder pain syndrome; ICCRN: Interstitial Cystitis Collaborative Research Network; ICCTG: The Interstitial Cystitis Clinical Trials Group; ICDB: Interstitial Cystitis Database study; ICINDEX: Interstitial cystitis symptom index (ICSI) and Problem Index (ICPI); IIEF: International index of erectile function; IPIP: The International Personality Item Pool; LONI: Laboratory of Neuroimaging; MAPP: Multidisciplinary Approach to the Study of Chronic Pelvic Pain; MASQ: Multiple ability self-report questionnaires; MCS: Mental health composite score; MRI/fMRI: Magnetic resonance imaging/functional magnetic resonance imaging; MSFS: Washington male sexual function scale; NIDDK: The National Institute of Diabetes, Digestive, and Kidney Diseases; NUAS: Non-urologic associated syndromes; OPPERA: Orofacial Pain Prospective Evaluation and Risk Assessment (https://www.oppera2.org/OPPERAII/Images/OPPERAIIRecruitmentBrochure.pdf); PANAS: Positive and Negative Affect Schedule; PCS: Physical functioning score; PPT: Pressure pain threshold; PROMIS: The NIH patient reported outcomes measurement information system; PSS: Perceived stress scale; RICE: RAND Interstitial Cystitis Epidemiology Study; RTES: Recent Traumatic Events Scale; SEAR: M & F: Self-esteem and relationship questionnaire; SF-12: The SF-12®: An Even Shorter Health Survey; SYM-Q: Symptom and health care utilization questionnaire; TATC: Tissue analysis and technology core; TMD: Temporomandibular disorders; Trans-MAPP EP: Trans-MAPP epidemiology and phenotyping study; UCLA: University of California at Los Angeles; UCPPS: Urological chronic pelvic pain syndromes; VB1,VB2: Voided bladder 1… 2.

## Competing interests

All authors participated in choosing the measures and variables which were collected in the MAPP network studies. All authors participated in the development of the MAPP network protocol. BD Naliboff, JQ Clemens, N Afari, CS Bradley, JW Griffith, P Hanno, BA Hong, JN Krieger, HH Lai, JR Landis, SK Lutgendorf, LV Rodriguez, AJ Schaeffer, S Sutcliffe, CC Yang, JW Kusek, Z Kirkali, EA Mayer, D Buchwald, NA Robinson, A van Bokhoven, and C Mullins declare no competing interests. DA Williams is a Consultant for Health Focus Inc. and has consulted for Pfizer. DJ Clauw has received grants from Pfizer, Cerephex, Lilly, Merck, Nuvo and Furest, and Consulting Fees and Honoraria from Pfizer, Cerephex, Lilly, Merck, Nuvo, Furest, Tonix, Purdue, Therauance, and Johnson & Johnson. DJ Klumpp declares ownership and equity interests in ProbioTx Inc, and Gold Coast Therapeutics Inc. GL Andriole is a Consultant for Augmenix, Bayer, Genomic Health, GlaxoSmithKline, and Myriad Genetics, and has received research grants from Johnson & Johnson, Medivation, and Wilex. KJ Kreder is a Consultant for Medtronic, Astellas, Symptelligence, and Tengion. MA Pontari is a Consultant for Lilly and Watson and has received Royalties from Up to Date. MS Lucia declares ownership of 3D Biopsy and has consulted for Myriad Genetics and Bayer Healthcare.

## Authors’ contributions

JRL, DAW and JQC wrote the initial draft manuscript. All authors read and approved the final manuscript.

## Pre-publication history

The pre-publication history for this paper can be accessed here:

http://www.biomedcentral.com/1471-2490/14/58/prepub
